# Development of a Deimmunized Bispecific Immunotoxin dDT2219 against B-Cell Malignancies

**DOI:** 10.3390/toxins10010032

**Published:** 2018-01-06

**Authors:** Joerg U. Schmohl, Deborah Todhunter, Elizabeth Taras, Veronika Bachanova, Daniel A. Vallera

**Affiliations:** 1 University of Minnesota Masonic Cancer Center, Section of Molecular Cancer Therapeutics, Therapeutic Radiology-Radiation Oncology, University of Minnesota, Minneapolis, MN 55455, USA; joerg.schmohl@med.uni-tuebingen.de (J.U.S.); todhu001@umn.edu (D.T.); taras003@umn.edu (E.T.); 2 Department for Hematology and Oncology, University Hospital of Tuebingen, Medical Department 2, 72076 Tuebingen, Germany; 3 Division of Hematology, Oncology, and Transplantation, Department of Medicine, University of Minnesota, Minneapolis, MN 55455, USA; bach0173@umn.edu

**Keywords:** diphtheria toxin, B-cell malignancies CD19, CD22, deimmunized, immunotoxin, The new dDT2219 targeted toxin combines potent anti-tumor cell activity with a reduced immunogenicity.

## Abstract

Diphtheria toxin (DT) related targeted toxins are effective in cancer treatment, but efficacy diminishes in time because of their immunogenic potential and/or former vaccinations. In order to overcome this limitation for DT2219, a promising bispecific targeted toxin which targets CD19 and CD22, we deimmunized the DT moiety, and thereby developed an exciting improved drug (dDT2219) which still has the potential to sufficiently target B-cell malignancies but also limits clearance because of its reduced immunogenicity. The DT moiety was modified by inducing point mutations in prominent positions on the molecular surface. The new engineered dDT2219 was tested for activity, efficacy, and specificity using functional assays, proliferation assays, and flow cytometry. Furthermore, 12 samples of Chronic Lymphatic Leukemia (CLL) patients were used to assess binding. Immunogenicity was determined using a BALB/c mouse model. dDT2219 was efficient and specific against B-cell malignancies such as Bukitt-Lymphoma cell lines Daudi and Raji. dDT2219 showed specific binding on targets and on CLL samples. Intraperitoneal vaccination of immune competent mice showed that even after multiple administrations with increasing doses, induction of neutralizing antibodies was significantly lower in the dDT2219 treated animal group. The new dDT2219 combines potent anti-tumor cell activity with a reduced immunogenicity. With regard to the frequent development of neutralizing antibodies after multiple administrations with immunotoxins, dDT2219 shows promise to overcome this limitation and thus might maintain effectiveness even after multiple treatment cycles.

## 1. Introduction

Immunotoxins are effective constructs in the treatment of malignancies. They consist of one or more binding sites which are capable of binding and killing tumor-associated antigen (TAA) expressing cells. The mechanism of tumor elimination depends on the linked peptide toxin. Diphtheria toxin (DT) containing targeted toxins (TTs) have proven effective in eliciting anti-cancer responses in animals as well in humans by using scFvs to target binding sites [1,2,3].

Several TTs are documented, using DT for tumor elimination. Denileukin diftitox is used for relapsed/refractory cutaneous T-cell lymphoma [1] and DTAT/DTAT13 against glioblastoma [2]. Both TTs were reported to have great potential in eliminating target cells of choice. Recently our study group revealed in a phase I clinical study the efficacy of DT2219 for mature or precursor B-cell lymphoid malignancies by targeting CD19 and CD22 [3].

DT toxin is a protein containing 535 amino acids. The functional domains consist of a C-terminal B domain and an N-terminal A domain. Whereas the B domain binds most eukaryotic cells and therefore has to be removed for human therapy, the A domain contains with 390 amino acids the catalytic enzyme that ADP-ribosylates elongation factor 2 (EF-2) and inhibits protein synthesis after internalization into the target cell which finally leads to apoptosis [4]

As frequently seen in treatment with TTs, immunogenicity represents a challenging problem. Multiple treatment courses lead to clinical production of anti-drug antibodies [5]. This was reported for DT2219 and for other DT containing immunotoxins in phase I and II clinical studies [3,6,7]. Furthermore, vaccination with diphtheria, tetanus, and pertussis at a young age ensures immunization against DT in a majority of patients. Thus, limitation of immunogenicity especially for DT containing TTs is a desirable goal, enabling multiple treatment courses without limitation in efficacy.

CD19 is a transmembrane protein and considered a pan-B-cell marker. In physiologic conditions, CD19 is involved in intrinsic signaling and modulation of the B-cell receptor. CD19 is expressed during the whole maturation process of B-cells, even in early stages [8], and thus is an important marker for diagnosis and classification of B-cell malignancies [9]. CD22 is a membrane-bound receptor expressed on B-cells. The fact that CD22 is also expressed during a majority of development stages and highly expressed on mature B-cells makes it a valuable marker for a variety of B-cell malignancies [10].

In this study, we replaced DT of the potent bispecific TT DT2219, capable of binding CD19 and CD22 alike, and formed dDT2219. The new construct contains a deimmunized form of DT. By mutating R, K, D, E, and Q amino acids, the new construct contains the modified DT toxin DT390 which promises to be less immunogenic compared to the parental form.

## 2. Results

### 2.1. Construction of dDT2219

**Figure 1:**
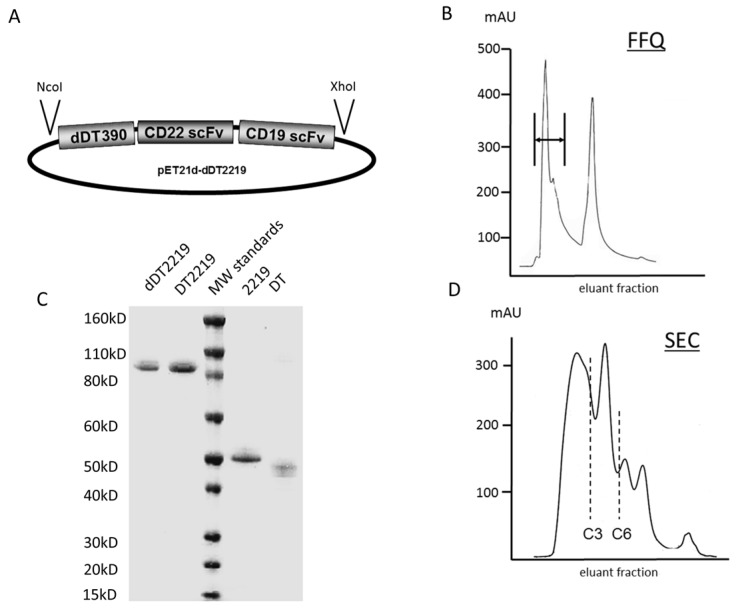
Construction and purification of dDT2219. (**A**) The full dDT2219 gene consists of a NcoI restriction site, the first 390 amino acids of the deimmunized DT molecule (dDT390), spliced to an anti-CD22 scFv, spliced to an anti-CD19 scFv and to the XhoI restriction site. (**B**) Q sepharose Fast Flow (FFQ) shows the absorbance trace for dDT2219. The arrow marks the product of interest. (**C**) Size comparison was performed via SDS-PAGE gel and Coomassie Blue staining. dDT2219, DT2219, molecular weight standard (MW standards), an anti-CD19 scFv spliced to an anti-CD22 scFv devoid of DT_390_ (2219) and diphtheria toxin (DT_390_) alone (DT]) were compared in size. dDT2219 is 97.5 kD in size. (**D**) Purity was over 90% after size exclusion column (SEC) purification (product is marked between C3 and C6 fraction).

In order to construct a deimmunized TT capable of targeting CD19 and CD22, dDT2219 was assembled. dDT2219 contains DNA fragments encoding deimmunized DT (dDT_390_), spliced to the V_H_ and V_L_ regions of an anti-CD22 and an anti-CD19 scFv. The active fragments are connected via an EASGGPE and an aggregate reducing linker (ARL) linker, forming dDT2219 (Figure 1A). Absorbance tracing for dDT2219 eluted from the FFQ ion exchange column as the first phase in drug purification using a three-step elution protocol is displayed in Figure 1B. The first peak eluted from the column represents the product of interest. SDS-PAGE gel (Figure 1C) and Coomassie Blue staining show purity after both ion exchange and size exclusion column purifications (Figure 1D). The product is over 90% pure and about 97.5 kD in size. To determine purity, the gel was scanned with a Beckman spectrophotometric gel-scanning accessory at 560 nm wavelength and analyzed with a Gel scan software module (Beckman Instruments, Fullerton, CA, USA).

### 2.2. Activity/Specificity of the Mutated dDT2219

**Figure 2:**
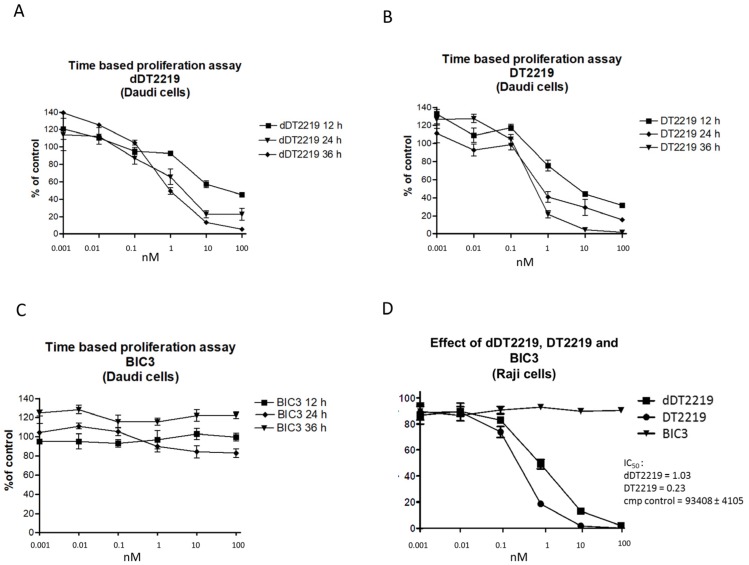
Activity and kinetics of dDT2219. The activity of (**A**) dDT2219, (**B**) DT2219, and (**C**) BIC 3 (a monospecific anti-CD3 scFv conjugated with DT_390_) was evaluated using Daudi cells. Comparison was performed after an incubation time of 12, 24, or 36 h with an increasing concentration of the drugs. In (**D**), a direct comparison was performed with a different lymphoma cell line (Raji). Data represent mean ± standard deviation of three independent experiments.

In order to study activity and kinetics of dDT2219 compared with the parental DT2219 form, we performed proliferation assays with CD19 and CD22 positive Burkitt’s lymphoma cell lines Daudi and Raji. Therefore, H^3^ Thymidine uptake of Daudi cells was evaluated after exposure to each respective drug (Figure 2A dDT2219, Figure 2B DT2219) for 12, 24, and 36 h in increasing concentrations (0.001, 0.01, 0.1, 1, 10, and 100 nM). Comparison showed that at higher concentrations (1–100 nM) after 24 and 36 h, a slightly increased percentage of activity in the DT2219 group was visible, whereas in our control group with exposure to BIC3 (bivalent scFv construct consisting of the catalytic and translocation domains of diphtheria toxin and two anti-CD3 scFv domains) no inhibition of proliferation at all time points was obvious. (Daudi cells do not express CD3) (Figure 2C). In order to enhance reproducibility, we used an additional Burkitt’s lymphoma cell line Raji. In a side-by-side proliferation assay using dDT2219, DT2219, and BIC3 for 72 h of incubation, an IC_50_ of 1.03 for dDT2219 and 0.23 for DT2219 was estimated. BIC3 again had no effect (Figure 2D).

**Figure 3:**
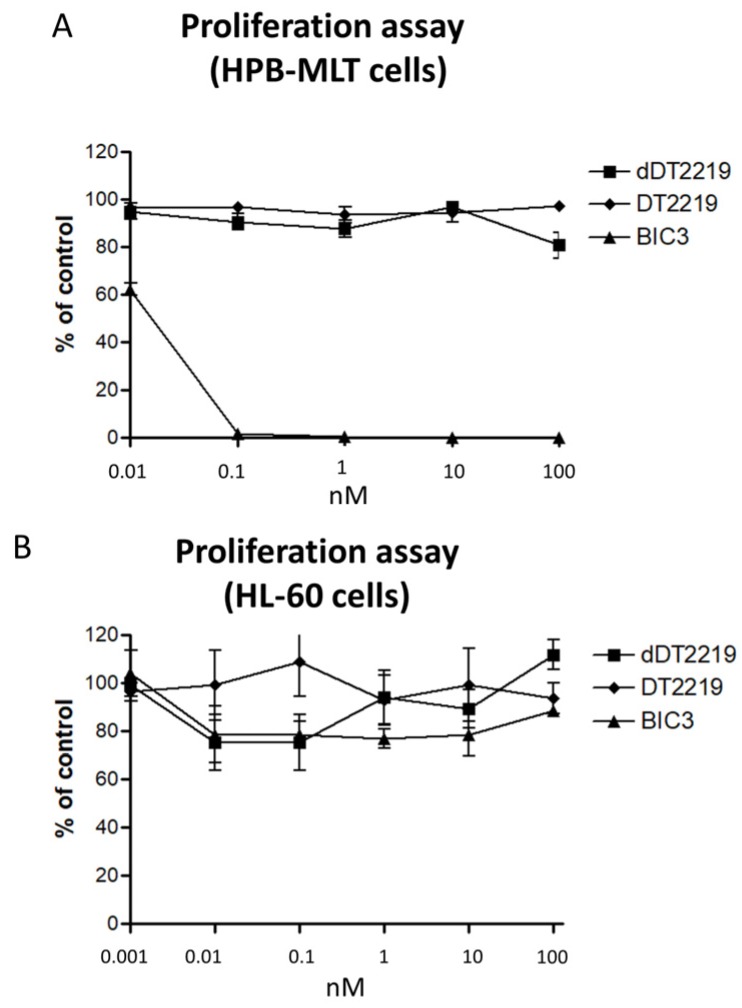
Specificity of target cell elimination. In order to evaluate specificity of dDT2219 (DT2219 and BIC3 are controls), proliferation assays were performed with (**A**) HPB-MLT cells and (**B**) HL-60 cells. Data represent mean ± standard deviation of three independent experiments.

For verification of specificity of dDT2219 (DT2219 and BIC as controls), we performed a proliferation assay with HPB-MLT cells (T-cell leukemia cell line expressing CD3 but not CD19 or CD22). dDT2219, DT2219, and BIC3 were tested using increasing concentrations. No effects after dDT2219 and DT2219 exposure were visible. Only BIC3 induced apoptosis (Figure 3A). For the acute promyeloid leukemia cell line HL-60 that is negative for CD22, CD19, and CD3, no inhibition after treatment was observed (Figure 3B).

### 2.3. Binding Characteristics of dDT2219

**Figure 4:**
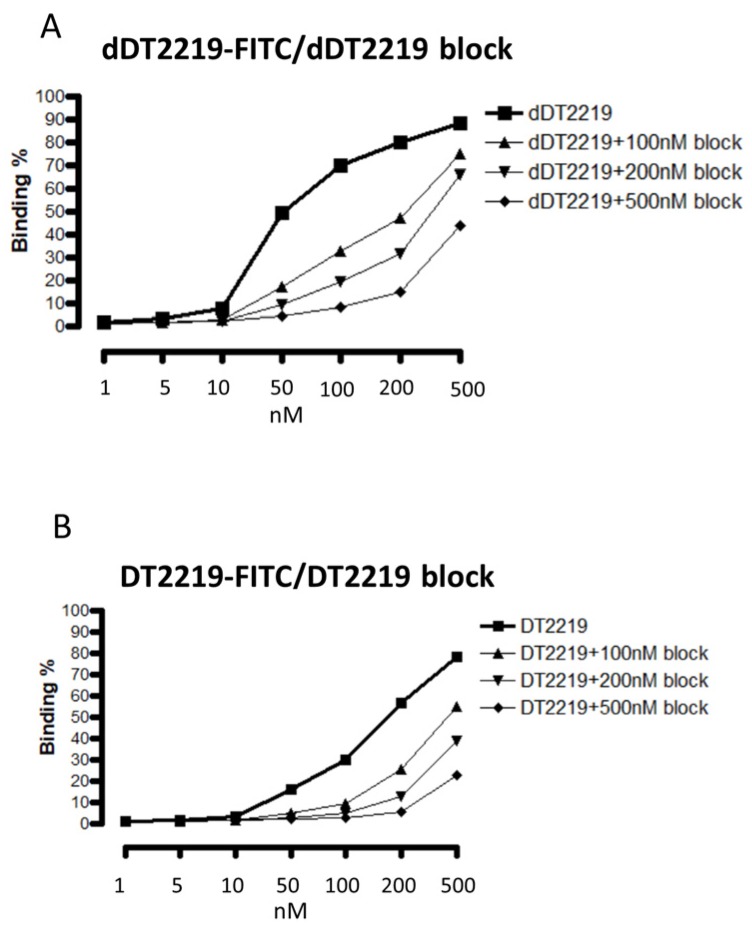
Evaluation of specific binding. Daudi cells were exposed to FITC labeled dDT2219 (**A**) or DT2219 (**B**) in varying concentrations. Furthermore, Daudi cells were again exposed to dDT2219-FITC and DT2219-FITC with the identical unlabeled drugs in 100, 200, and 500 nM, respectively.

Binding and blocking characteristics were performed using flow cytometry. FITC labeled dDT2219 (Figure 4A) and DT2219 (Figure 4B) were tested with increasing concentrations (1, 5, 10, 50, 100, 200, 500 nM) incubated with Daudi cells. Binding capability was dose dependent (>85% for dDT2219 and >75% for DT2219). The same cells were treated with the same doses of dDT2219 or DT2219 and also exposed to 100, 200, or 500 nM of unlabeled dDT2219 or DT2219. The deimmunized as well as the parental unlabeled drug showed a sufficient binding and consecutive and dose dependent blocking of the FITC labeled drugs, as seen in a reduction of fluorescence intensity.

**Figure 5:**
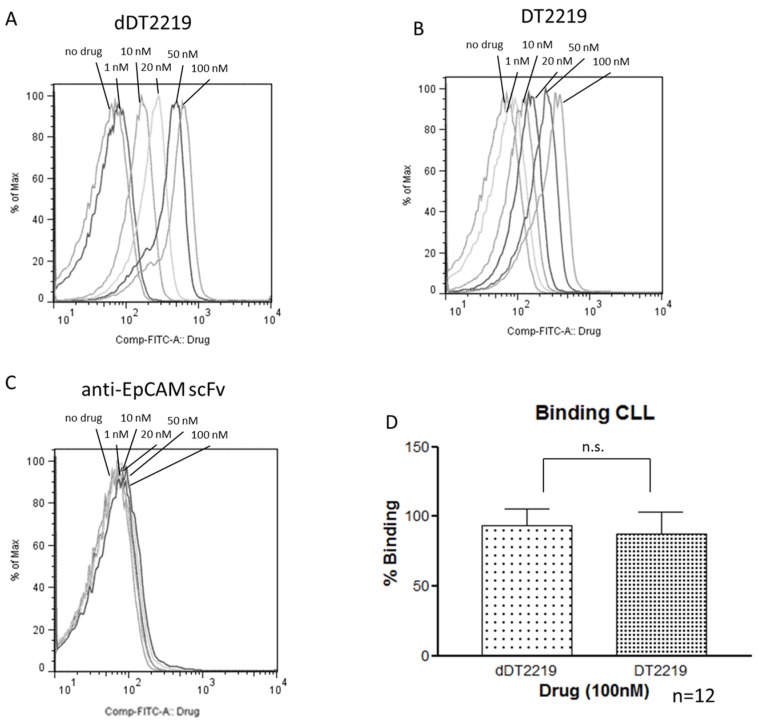
Binding in Chronic Lymphatic Leukemia (CLL). Patient derived CLL samples were exposed to FITC labeled dDT2219, DT2219, and an anti-EpCAM scFv (control). (**A**–**C**) show results after exposure of increasing concentrations of the respective drugs as labeled. (**D**) Patient derived CLL samples were exposed to FITC labeled dDT2219 or DT2219. Data represent mean ± standard deviation of 12 independent experiments.

In order to verify the binding capability in a clinical context, we used samples of Chronic Lymphatic Leukemia (CLL) patients and compared respective binding of dDT2219 and DT2219. Therefore, samples were exposed to increasing concentrations (no drug, 1, 10, 20, 50, 100 nM) of FITC labeled dDT2219 or DT2219. After gating on the CLL population, a dose dependent increase in binding was seen for both constructs (Figure 5A,B). A FITC labeled anti-EpCAM scFv was used as a negative control (Figure 5C) and showed no binding. In a direct comparison between dDT2219 and DT2219 each patient sample was exposed to 100 nM of dDT2219 or DT2219. The mean fluorescence (representing binding) seen in all 12 CLL samples was 93% and 88%, with no significant differences between the groups (*p* = 0.25) (Figure 5D), thus implying identical binding characteristics.

### 2.4. Immunogenicity in Mice

**Figure 6:**
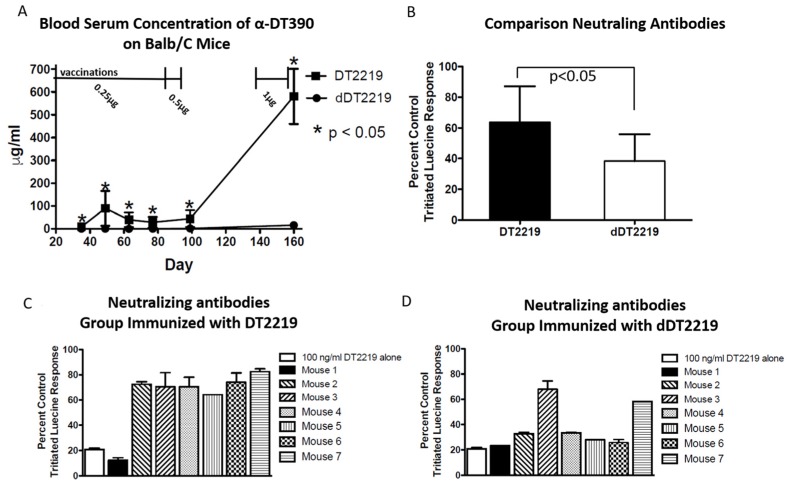
Neutralizing antibodies. Fourteen BALB/c immune competent mice were divided into two groups with seven mice respectively. Both groups were intraperitoneally vaccinated with dDT2219 (experimental group) or DT2219 (control group) (immunized weekly for 12 weeks with 0.25 μg protein, and then boosted with 0.5 μg protein weekly (two immunizations total), rested for 6 weeks, followed by weekly injections of 1 μg protein for 3 weeks) and bled on days 21, 35, 49, 63, 77, 99, and 160. (**A**) Sera were evaluated by performing an ELISA detecting anti-DT390 antibodies. (**B**) To evaluate functionality of detected antibodies, a neutralization assay was performed using Raji targets. (**C**,**D**) shows a direct comparison between the respective mice in the two groups.

To determine if neutralizing antibodies develop in the sera of immunized mice after repetitive exposure to dDT2219, BALB/c mice were divided into two groups with seven animals/group (experimental and control group). Both groups were immunized simultaneously and boosted weekly, as described in the methods. Both groups were treated with an equal concentration of dDT2219 or DT2219, as described above. On all evaluated days, sera of the dDT2219 group showed a significantly lower (*p* < 0.05) antibody induction, seen in an ELISA detecting anti-DT390 (Figure 6A). Even after four boosts with 1 µg of the respective drug at the end of the experiment, the dDT2219 group showed a significantly lower immunization.

In order to specify if detected antibodies indeed neutralize dDT2219 or DT2219, we performed a neutralization assay using Raji targets and sera of both animal groups on day 160. Significantly lower amounts of neutralizing antibodies (*p* < 0.05) were found in the dDT2219 group compared to the control group vaccinated with DT2219 (Figure 6B). Six of seven mice, (mice 2 to 7), developed a high titer of neutralizing antibodies (Figure 6C) after DT2219 vaccination, whereas only two of seven mice, (mice 3 and 7) developed neutralizing antibodies in the group immunized with dDT2219 (Figure 6D).

## 3. Methods

### 3.1. Construction of dDT2219

The dDT2219 gene was synthesized using assembly PCR. The fully assembled gene (from 5′ end to 3′ end) consisted of a NcoI restriction site, an ATG initiation codon, the first 390 amino acids of the mutated and deimmunized DT molecule (DT_390_), the 7 amino acid EASGGPE linker, the V_L_ and V_H_ regions of an anti-CD22 scFv, a GGGGS linker, the V_L_ and V_H_ regions of an anti-CD19 scFv, and a XhoI restriction site. The V_L_ and V_H_ gene of each scFv were joined by a linker (GSTSGSGKPGSGEGSTKG) that we designated as aggregate reducing linker (ARL). The final 1755bp NcoI/XhoI target gene was spliced into the pET21d expression vector under control of an isopropyl-b-d-thiogalactopyranoside (IPTG) inducible T7 promoter. DNA analysis was used to verify that the gene was in correct sequence (Biomedical Genomics Center, University of Minnesota, Minneapolis, MN, USA). To create a deimmunized drug, DT2219 was mutated using the QuickChange Site-Directed Mutagenesis Kit (Stratagene, La Jolla, CA, USA) and site-specific mutations were confirmed by DNA sequencing. The following amino acids were changed to deimmunize DT_390_: amino acid (aa) 125 changed from K to S; aa 173 R to A; aa 184 Q to S; aa 227 K to S; aa 245 Q to S; aa 292 E to S; and aa 385 K to G. Mutations were proved to be critical for immunogenicity [11].

### 3.2. Inclusion Body Isolation, Refolding, and Purification

*Escherichia coli* strain BL21 (DE3) (plasmid transfected) (Novagen, Madison, WI, USA) was used for protein expression. Bacteria were cultured overnight in 800 ml Luria broth, containing 50 mg/ml carbenicillin. The gene expression was induced via addition of IPTG (Fischer Biotech, Fair Lawn, NJ, USA) after culture reached an optical density (OD) 600 of 0.65. Two hours after induction, grown bacteria were harvested and centrifugated. The pellet was homogenized in a solution buffer (50 mM tris, 50 mM NaCl, and 5 mM EDTA pH 8.0) and then sonicated and again centrifuged. Extraction of the pellet was performed using 0.3% sodium deoxycholate, 5% Triton X-100, 10% glycerin, 50 mmol/L Tris, 50 mmol/L NaCl, 5 mmol/L EDTA (pH 8.0) and washed. Purification was described previously [12,13]. Homogenized inclusion bodies were dissolved and proteins refolded. Purification was performed using an ion exchange chromatography (Q sepharose Fast Flow, Sigma-Aldrich, St. Louis, MO, USA) with a continuous gradient and Superdex 200 sizing columns.

### 3.3. Tissue Cultures

The following cell lines were obtained from the American Type Culture Collection: Burkitt’s lymphoma cell lines Daudi and Raji, T-cell leukemia HPB-MLT, human promyelocytic leukemia cell line HL-60. All cell lines were grown in suspension [14]. Cells were maintained in RPMI 1640 (supplemented with 10% fetal serum).

### 3.4. Proliferation Assay/Blocking Assay

In order to evaluate drug efficacy, (2 × 10^4^) target cells were plated into a 96-well round bottom plate and suspended in RPMI supplemented with 10% fetal bovine serum, 2 mM L-glutamine, 100 U/mL penicillin, and 100 µg/mL streptomycin. DT2219, dDT2219, or BIC3 (bivalent scFv construct consisting of the catalytic and translocation domains of diphtheria toxin and two anti-CD3 scFv [15]) was added in different concentrations. Assays were performed in triplicate. For time dependent assays, cells were incubated for 12, 24, and 36 h. For regular proliferation assays, 72 h of incubation was performed at 37 °C, 5% CO_2_. Cells were then incubated with one µCi [methyl-3H]-thymidine (GE Healthcare, Buckingham, UK) per well for eight hours and harvested onto glass fiber filters and washed. After a drying step, filters were counted for ten minutes in a standard scintillation counter. Data were analyzed using Prism 4 (GraphPad Software, Inc., La Jolla, CA, USA) and were presented as “percent control response” calculated by dividing the counts per minute (CPM) of untreated cells by the CPM of the immunotoxin-treated cells (×100).

In order to conduct specificity, we also performed blocking studies. The amount of 100, 200, and 500 nM of 2219 (the bispecific anti-CD19 and anti-CD22 scFv construct devoid of DT_390_) was added to the respective amounts of either DT2219 or dDT2219 [16] and incubated with target cells for 37 °C, 5% CO_2_. After incubation, H^3^-thymidine uptake was measured as described above.

### 3.5. Patient Samples and Binding Assay

Twelve patient samples from CLL patients were collected at the University of Minnesota which were diagnosed with standard procedures. All samples were collected after patients declared written informed consent in accordance with the ethical standards of the institutional review board and with the Helsinki Declaration of 1975, as revised in 2013. Samples were cryopreserved and stored according to standard procedures at the Masonic Cancer Center’s Translational Therapy Laboratory until use. For evaluation of binding, 4 × 10^5^ peripheral blood nuclear cells (PBMCs) of the respective samples were washed and incubated at 4 °C with no, 1, 10, 20, 50, and 100 nM of fluorescein isothiocyanate (FITC)-labeled constructs: dDT2219, anti-EpCAM scFv (negative control), DT2219. After 30 min of incubation at 4 °C, cells were washed in 1× PBS and stained with APC Cy 7 conjugated anti-CD20 monoclonal antibody (mAb)^a^, Pacific blue conjugated anti-CD5^a^ mAb, PE-CF592 conjugated anti-CD3 mAb^b^ from; ^a^ (BioLegend, San Diego, CA, USA) and ^b^ (BD Biosciences, San Jose, CA, USA) according to [17]. After incubation for 15 min, (4 °C), cells were washed with 1× PBS and fluorescence intensity was evaluated by FACS analysis using a LSRII flow cytometer (BD Biosciences, San Jose, CA, USA).

### 3.6. Detecting Anti-Toxin Antibodies in Mice

Our assay to detect IgG anti-toxin antibodies was previously reported [11]. Briefly, immunocompetent normal BALB/c mice (NCI) were intraperitoneally immunized with non-mutated DT2219 (*n* = 7 mice) or mutated DT2219 (dDT2219) (*n* = 7 mice) (experiment registration number DAV529). Mice were immunized weekly for 12 weeks with 0.25 μg protein, followed by immunizations with 0.5 μg protein weekly (two immunizations total), rested for 6 weeks, followed by weekly injections of 1 μg protein for 3 weeks. The experiment ended after 160 days. Blood was drawn on days 21, 35, 49, 63, 77, 99, 160. A standard ELISA assay was used in which recombinant DT390 was adhered to the plate. Test serum from the immunized mice was then added followed by the detection antibody and anti-mouse IgG peroxidase (Sigma-Aldrich, St. Louis, MO, USA). Plates were developed with *o*-phenylenediamine dihydrochloride (Thermo Scientific, Rockford, IL, USA) for 15 min at room temperature. The reaction was stopped with the addition of 2.5 M H_2_SO_4_. Absorbance was read at 490 nm and the final concentration was determined from a standard curve using a mouse monoclonal antibody to diphtheria toxin (Abcam Inc., [11D9], Cambridge, MA, USA). All samples and standards were tested in triplicate. Experiments were performed in accordance to the University of Minnesota Animal Care and Use Committee (IACUC), the project identification code is 1609-34115A, protocol title is “Immunotoxins in Bone Marrow Transplantation”, and approval date is 5 December 2017.

### 3.7. Detecting Neutralizing Antibodies in Mice

To detect neutralizing antibodies, 90% serum from immunized mice was added to cells, and treated with a known inhibitory concentration of DT2219 and dDT2219 (1000 ng/mL). Proliferation assays were then carried out as described above. For detection of serum IgG, anti-toxin content ELISA Assay was used.

### 3.8. Statistical Analyses

Data are presented as mean ± standard deviation. For evaluation of differences between the groups, Student’s *t*-test or one-way-ANOVA was used. Analysis and presentation of data was done with GraphPad prism 5 (GraphPad Software Inc., La Jolla, CA, USA).

## 4. Discussion

To effectively use TTs synthesized by bacteria, strategies need to be found to reduce their immunogenic potential in order to facilitate multiple administrations. The original contribution of this work is the first report of the improved bispecific TT dDT2219, engineered by using a modified A domain of DT [11] and the corpus of the established and effective drug DT2219 [3]. dDT2219 showed the same activity profile as DT2219 but had, in contrast, a significantly lower immunogenic potential. The deimmunized form of DT2219 might facilitate long-term administrations without limitation of anti-drug antibodies seen in the parental form [3], which enables longer treatment cycles to effectively eliminate cancer cells.

Over the past years, evidence has mounted, indicating that TTs can serve a major role in eliminating tumor cells. As reported for a variety of solid [18] and hematologic malignancies [1], DT based TTs such as Denileukin diftitox (DAB389IL-2) against relapsed/refractory cutaneous T-cell lymphoma [1], DTAT and DTAT13 or DTIL13 against glioblastoma [2], and DT2219 against mature and precursor B-cell lymphoid malignancies [3] have proven to be effective in vivo. However, besides reported success in treatment, the use of DT-containing constructs is still problematic. Such immunogenic agents induce anti-drug antibodies during the treatment cycles. Studies of Denileukin diftitox revealed detectable anti-DT antibodies in 32% of 60 patients before initiation of treatment and after two courses of treatment, all except one patient developed anti-toxin antibodies [18]. Recently, DT2219 was used in a phase I study with patients suffering from refractory B-cell malignancies. In a total of 25 patients, neutralizing antibodies were found in 30% after four courses of administration and in all patients treated with ≥40 mg/kg/dose. Authors implied that immunogenic potential remains a fundamental problem of DT containing constructs. Furthermore, clinical studies using pseudomonas toxin related TTs induce neutralizing antibodies in a high titer after administration too [19,20,21]. Thus, immunogenicity is a problem for other toxins as well.

Approaches to limit immunogenicity have been investigated and include different strategies such as the use of polyethylene glycol (PEG) polymers, and RNases, which can be conjugated to bioactive drugs (reviewed in [22,23]). However, these approaches have not reached practical clinical status and alternative approaches are mandated. Other approaches include co-treatment with B-cell depletive agents such as rituximab. However, even after administration of rituximab and a targeted toxin (LMB-1) against Lewis Y antigen-positive tumors, no suppression in human antibody response to the applied toxin was observed [24].

A promising alternative was presented by Onda and Pastan by showing a way to shed the toxin structure (PE38) via identification and modification of immunogenic residues [25]. Based on this strategy to mutate R, K, D, E, and Q amino acids, a deimmunized version of DT was lately created by our study group which showed a remarkable reduction in anti-toxin induction compared to its parental form [11]. This paper describes our efforts to combine the deimmunized form of DT (DT_390_) with the bispecific TT DT2219. We used a mouse model for testing immunogenicity because there is evidence that humans and mice recognize the same epitopes of foreign origin, leading to induction of antibodies [26,27,28]. Our data revealed that dDT2219 is indeed deimmunized and shows low immunogenicity even after multiple injections and repetitive administrations. Results were not related to clearance differences since unpublished pharmacologic studies showed that DT2219 and dDT2219 were cleared similarly with half-lives ranging from 49 to 132 min with no statistical difference.

The importance of surface receptors such as CD19 and CD22 to treat B-cell malignancies are obvious considering multiple successful constructs proved to perform sufficient tumor elimination. Unconjugated mouse anti-CD19 antibodies (CLB-CD19) were used to treat low-grade non-Hodgkin’s lymphoma [29]. An immunotoxin (Anti-B4-bR) targeting CD19 was used against relapsed B-cell non-Hodgkin’s lymphoma (NHL) [30] and the CD19 and CD3 binding bispecific T-cell engager (Blinatumomab) was used to eliminate refractory B-precursor acute lymphoblastic leukemia [31]. All studies revealed efficacy in cancer elimination. Furthermore, efficacy of targeting CD22 was proved for the anti-CD22 antibody (Epratuzumab) which was used against B-NHL [32] and the CD22 targeting immunotoxins to treat B-malignancies [33]. However, there is evidence that targeting both receptors alike might be more effective than using monospecific binding sites, which implies an advantage of a construct binding both ligands such as DT2219 [3] and dDT2219. Studies show that bivalent targeting with DT2219 is superior to monovalent targeting and leads to a higher binding capability and consecutively improved internalization of the toxin [34,35].

Another advantage can be seen in hematopoietic malignancy studies including CLL, Acute myeloid leukemia, B-Acute lymphatic leukemia (ALL), lymphoma, and T-ALL. A subcategorization was possible in these diseases, dividing tumor cell expression profile of CD19 and CD22 into those cases with high expression of both markers, low levels of both markers, and of cases with high expression of one marker and low expression of the other [36]. Targeting both markers could have an advantage for elimination of malignant cells and might address drug resistance via shedding or downregulation of one receptor as a mechanism of tumor escape. Success of a bispecific TT working with this principle was recently assessed in a phase I clinical study. The bispecific immunotoxin DT2219 was tested in 25 patients suffering from mature and precursor B-cell lymphoid malignancies which expressed CD19 and/or CD22 after an intensive pretreatment (median prior chemotherapy lines, 8 failed hematopoietic transplantations). Besides a good safety profile, a limited patient number achieved high dose treatment clinical responses in the tested cohort [3].

Taken together, we deimmunized DT2219, a potent targeted toxin against B-cell malignancies. The new construct showed effective tumor elimination in vitro demonstrated in two different Burkitt-lymphoma cell lines. No toxic effect for cell lines not expressing CD22 or CD19 was documented. The new drug showed the same binding capacity to CLL as its parental form and a significantly lower induction of neutralizing antibodies in immunocompetent BALB/c mice.

DT2219 has proven to be promising in a phase 1 clinical study [3] inducing complete and partial responses. Immunogenicity responses due to prior DT immunization or drug therapy have presented a problem, but evaluation of DT2219 is still underway in phase 2 clinical studies. If in the final analysis, data necessitate a need for a less immunogenic therapy, dDT2219 will provide an important clinical option. Modified dDT2219 may represent a potential solution not only for immunogenic DT2219, but other DT-based drugs as well.
